# Supplementary material for: An evaluation of age-varying genetic effects underlying body-mass index and blood pressure in the UK Biobank
Source: PLoS Genet. 2026 Mar 20;22(3):e1012080. doi: 10.1371/journal.pgen.1012080 (PMC13029756; doi:10.1371/journal.pgen.1012080)
Supplement: S1 Note — (DOCX) [file pgen.1012080.s026.docx]

## Supplementary Note 1:

## Descriptive overview of effect size differences in our age-stratified GWAS results.

We present visual comparisons of the effect estimates derived for discovery SNPs between distant age-strata to help highlight examples of putative age-varying SNP effects. We focused on comparisons between the youngest (40-41 years), middle (54-55 years) and oldest strata (68-69 years). The aim of this assessment was to help elucidate whether there may be evidence for non-linear peaks in association in the middle age-period and provide a general overview on the extent of variation between ages.

In general, many GWAS effect estimates exhibited a high degree of consistency between age periods. The SNP effect on the phenotype derived in our GWAS analyses correspond to an additive model whereby β > 0 is interpreted as a directionally positive effect on the trait. Scatter plots depicting pairwise effect estimate comparisons are provided to help identify individual SNPs with the largest differences in effect size, and where the direction of the effect differed between age periods, though we note that many of these are likely be attributed to sampling error rather than a true difference in effect direction. Additional forest plots were generated depicting SNPs identified based on non-overlapping confidence intervals between age periods. As exemplar, we note the plots depicting the comparisons between the youngest (40-41 years) and middle (54-55 years) strata. This identified 9 SNPs for BMI, 19 SNPs for PP, 20 SNPs for SBP and 8 SNPs for DBP (**S1ii-S2ii and S3ii-S4ii Figs**). Similar trends were observed when cross-comparing between other time periods, i.e. the youngest (40-41 years) vs oldest (68-69 years) group (**S3-S6 Figs**), and middle (54-55 years) vs oldest (68-69 years) group (**S5-S12 Figs**).

All plots are structured such as plot (i) Scatter plot depicting the relationship between effect estimates derived between age periods. The dashed black line indicates the line of equality between groups. Plot (ii) Forest plot depicting SNPs with non-overlapping confidence intervals (CIs) between age periods, and the overall discovery effect.
