# Supplementary material for: An evaluation of age-varying genetic effects underlying body-mass index and blood pressure in the UK Biobank
Source: PLoS Genet. 2026 Mar 20;22(3):e1012080. doi: 10.1371/journal.pgen.1012080 (PMC13029756; doi:10.1371/journal.pgen.1012080)
Supplement: S1 Fig — (PDF) [file pgen.1012080.s027.pdf]

Comparison 1: between youngest (age 40-41) and middle (age 54-55) age groups.

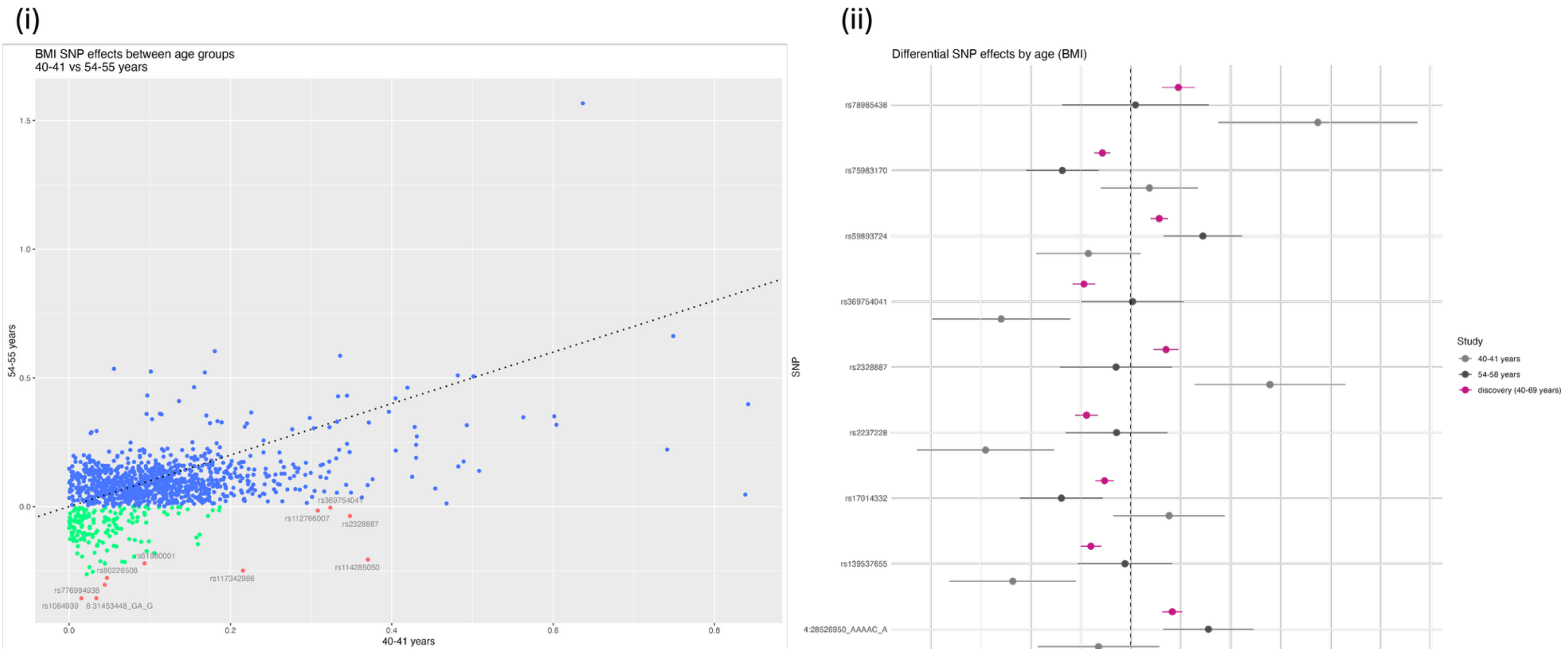

S1 Fig Comparison of GWAS effect estimates between Stratum 1 (40-41 years) and Stratum 8 (54-55 years) for BMI. *(i) Scatter plot depicting the relationship between effect estimates derived between age periods. The dashed black line indicates the line of equality between groups. (ii) Forest plot depicting SNPs with non-overlapping confidence intervals (CIs) between age periods, and the overall discovery effect.*
