# Supplementary material for: An evaluation of age-varying genetic effects underlying body-mass index and blood pressure in the UK Biobank
Source: PLoS Genet. 2026 Mar 20;22(3):e1012080. doi: 10.1371/journal.pgen.1012080 (PMC13029756; doi:10.1371/journal.pgen.1012080)
Supplement: S2 Fig — (PDF) [file pgen.1012080.s028.pdf]

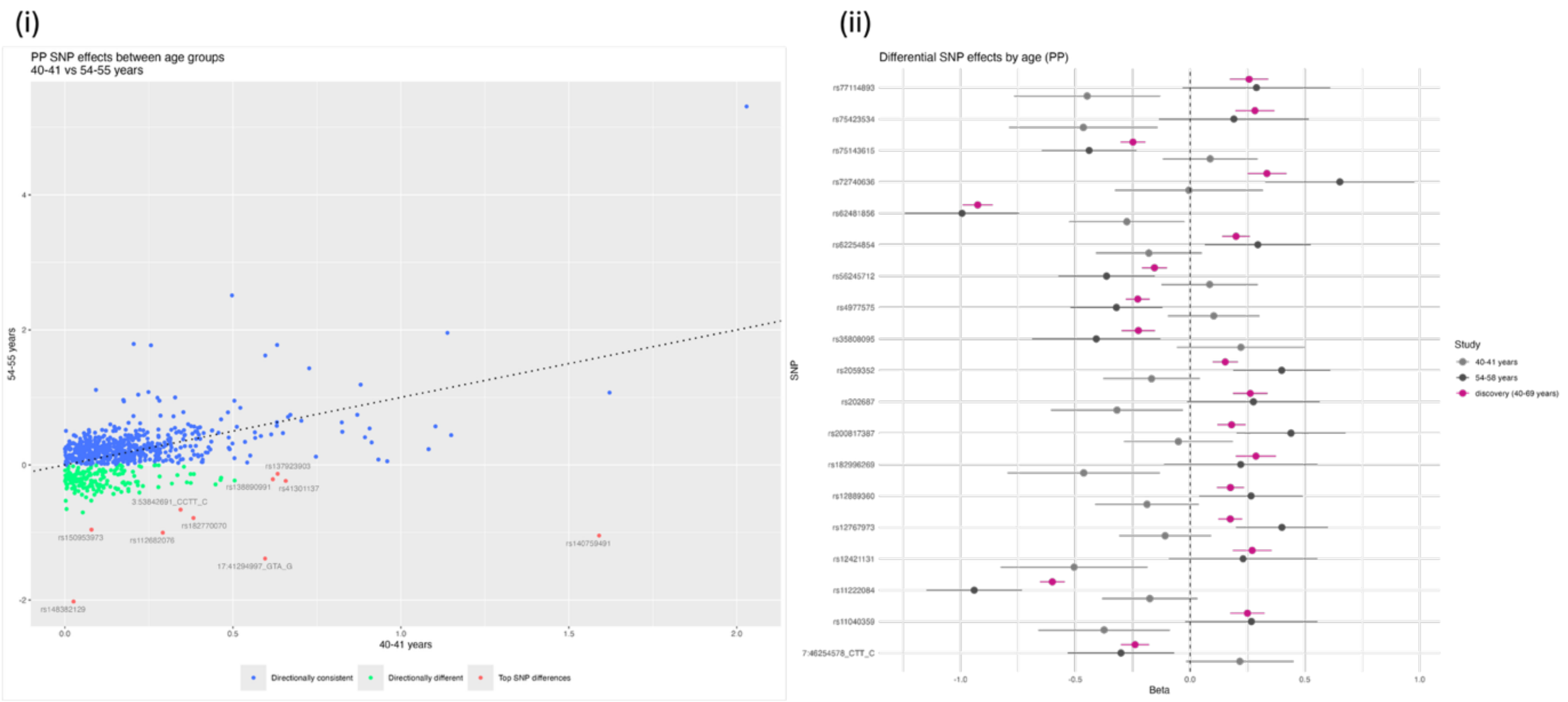

S2 FigComparison of GWAS effect estimates between Stratum 1 (40-41 years) and Stratum 15 (54-55 years) for PP.

(i) Scatter plot depicting the relationship between effect estimates derived between age periods. The dashed black line indicates the line of equality between groups. (ii) Forest plot depicting SNPs with non-overlapping confidence intervals (CIs) between age periods, and the overall discovery effect.
