# Supplementary figures and images for: An evaluation of age-varying genetic effects underlying body-mass index and blood pressure in the UK Biobank
Source: PLoS Genet. 2026 Mar 20;22(3):e1012080. doi: 10.1371/journal.pgen.1012080 (PMC13029756; doi:10.1371/journal.pgen.1012080)

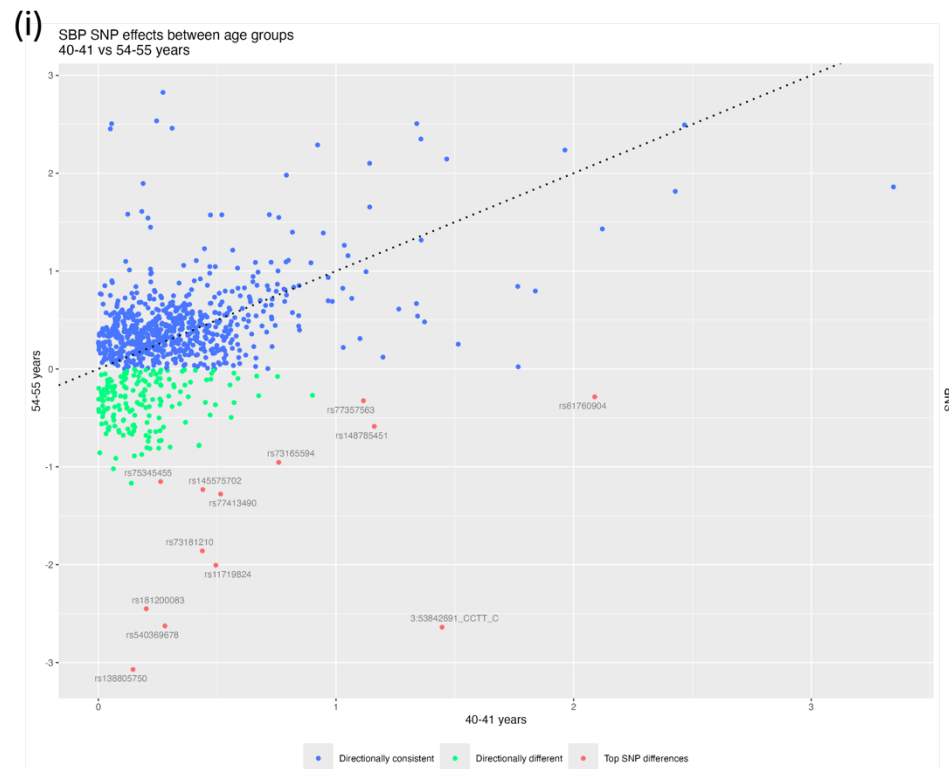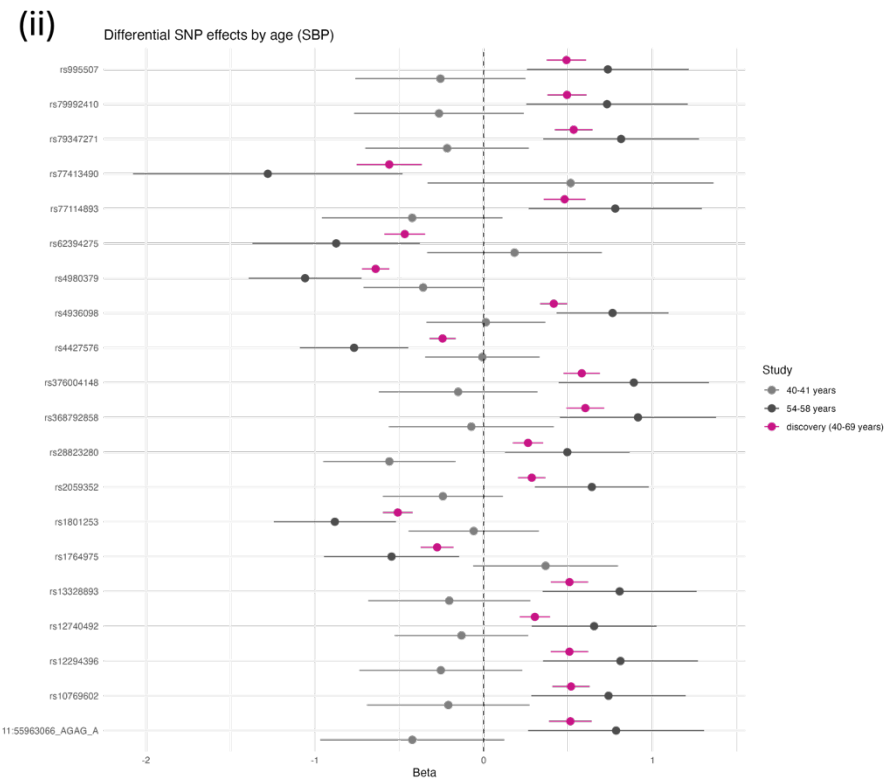

**S3 Fig Comparison of GWAS effect estimates between Stratum 1 (40-41 years) and Stratum 8 (54-55 years) for SBP.**

Supplement: S3 Fig — (PDF) [file pgen.1012080.s029.pdf]

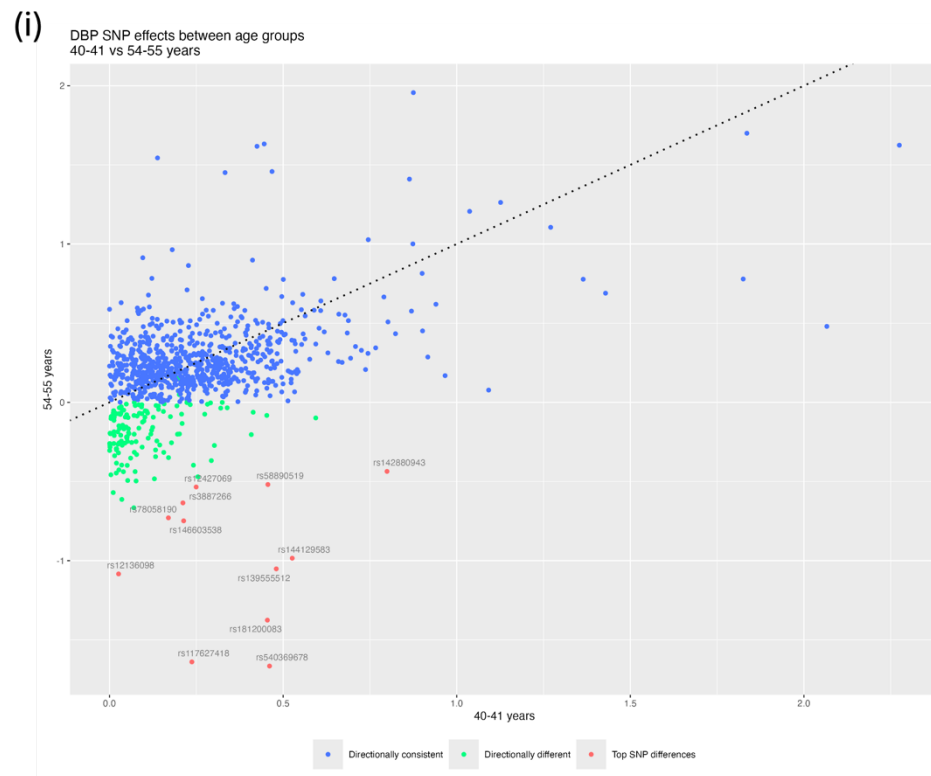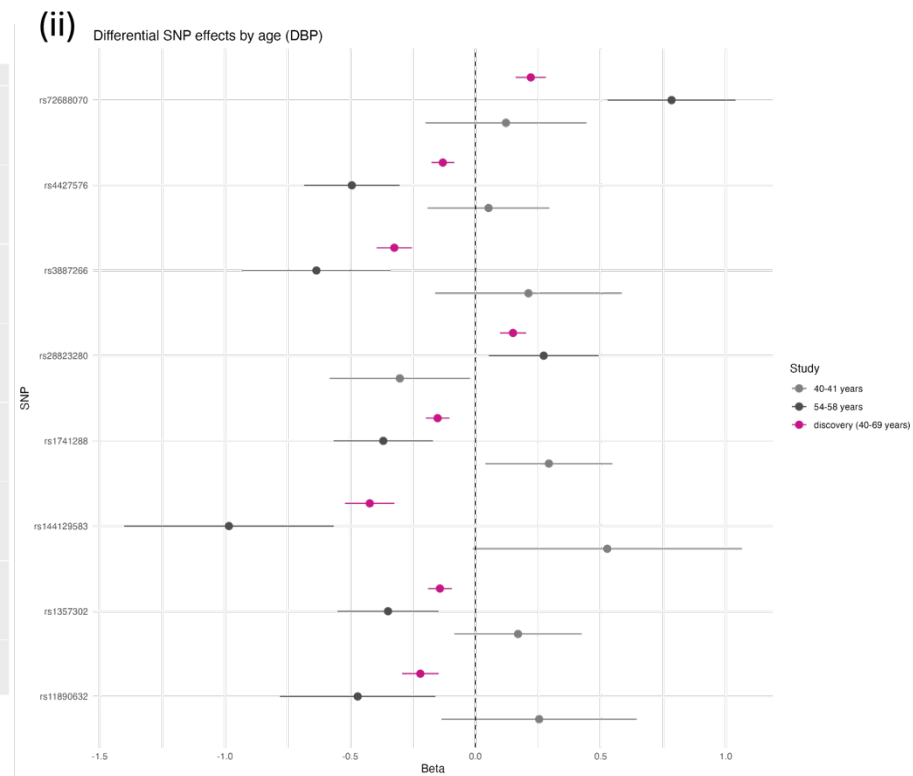

**S4 Fig Comparison of GWAS effect estimates between Stratum 1 (40-41 years) and Stratum 8 (54-55 years) for DBP.**

Supplement: S4 Fig — (PDF) [file pgen.1012080.s030.pdf]

(i)

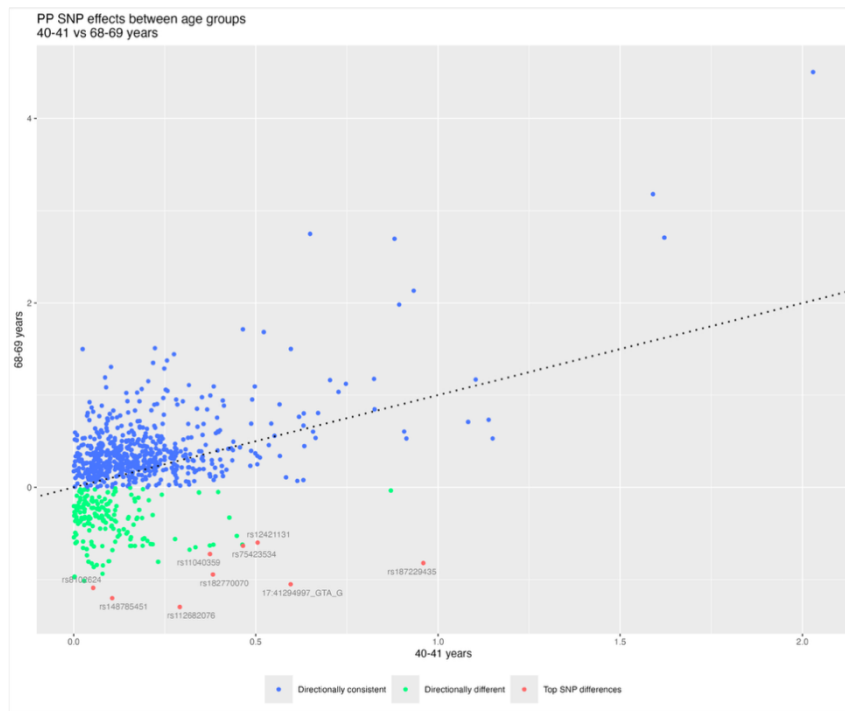

(ii)

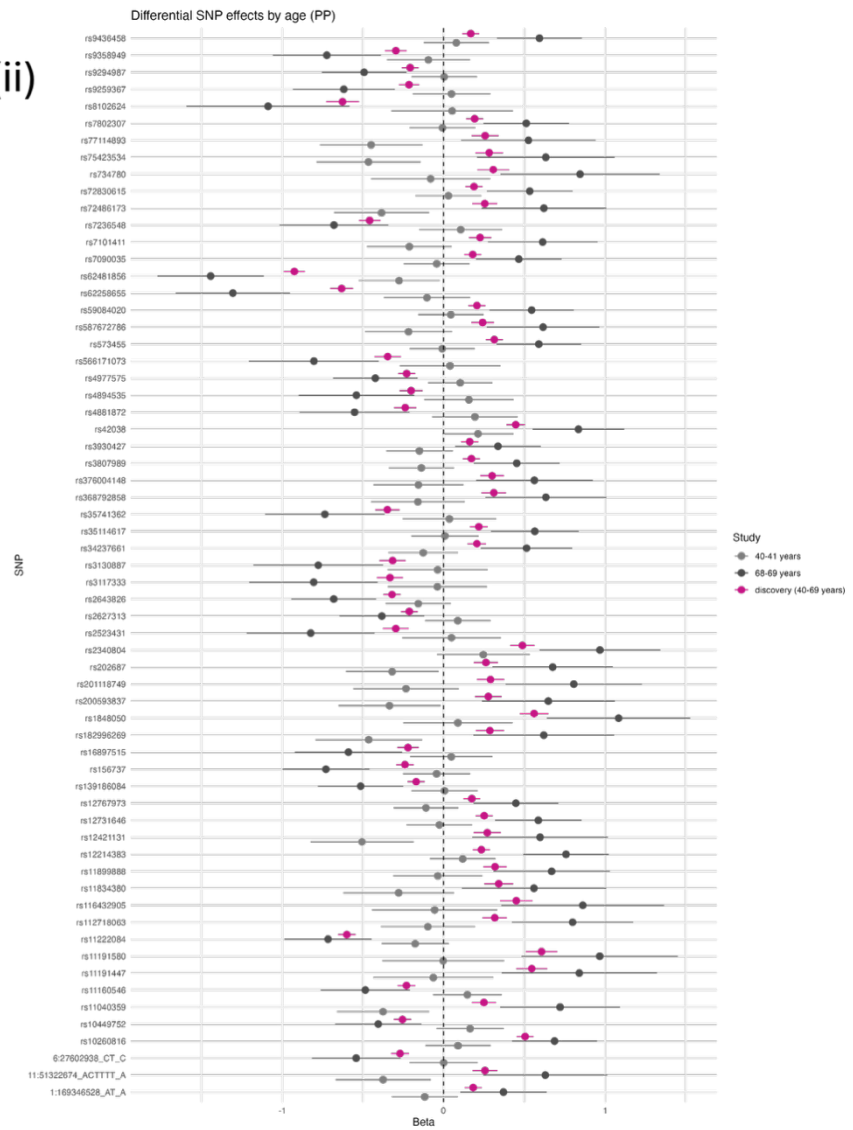

S6 Fig Comparison of GWAS effect estimates between Stratum 1 (40-41 years) and Stratum 15 (68-69 years) for PP.

Supplement: S6 Fig — (PDF) [file pgen.1012080.s032.pdf]

(i)

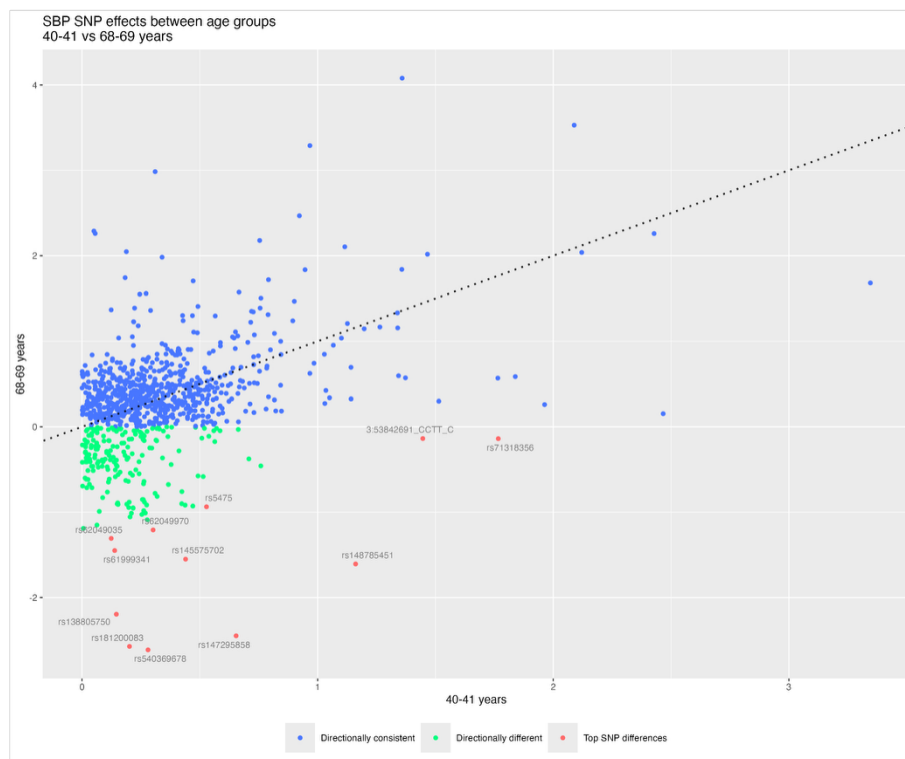

**S7 Fig Comparison of GWAS effect estimates between Stratum 1 (40-41 years) and Stratum 15 (68-69 years) for SBP.**

(ii)

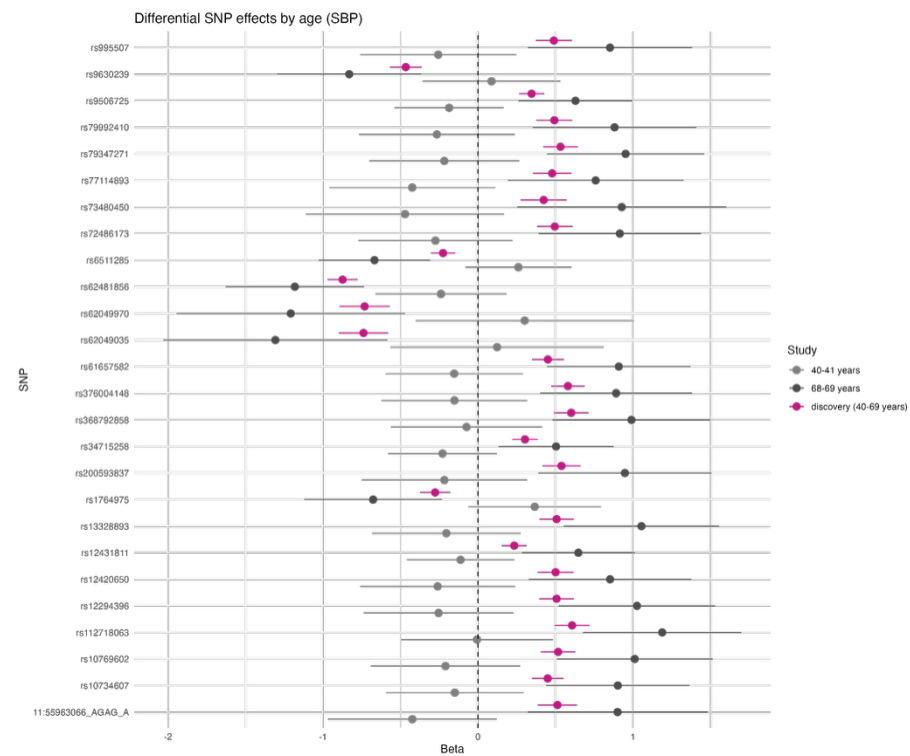

Supplement: S7 Fig — (PDF) [file pgen.1012080.s033.pdf]

(i)

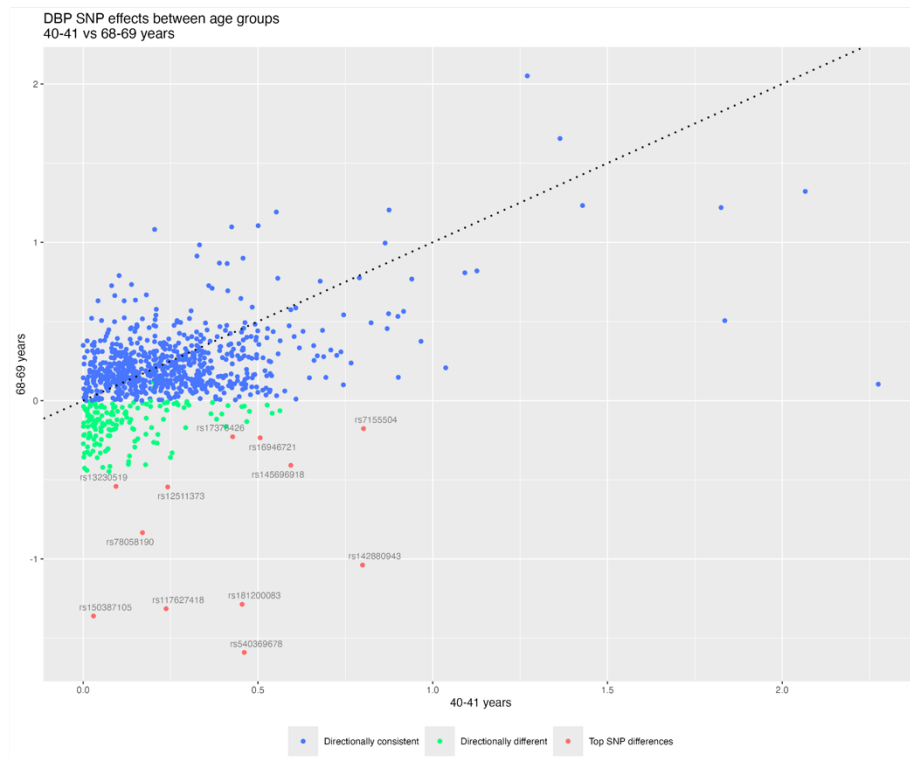

(ii)

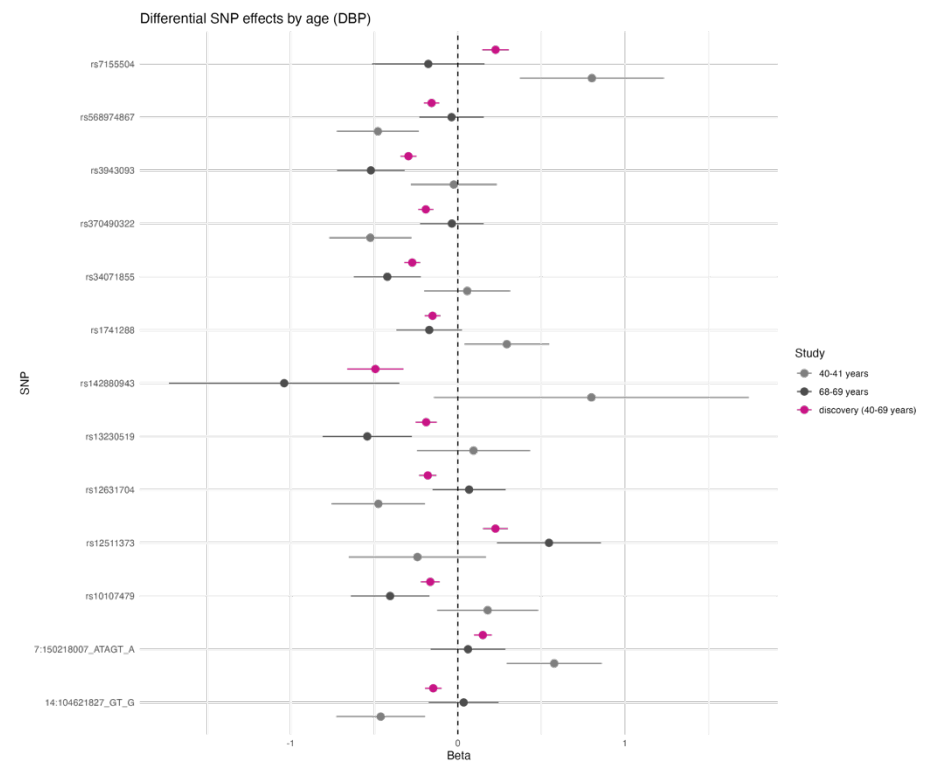

**S8 Fig Comparison of GWAS effect estimates between Stratum 1 (40-41 years) and Stratum 15 (68-69 years) for DBP.**

Supplement: S8 Fig — (PDF) [file pgen.1012080.s034.pdf]

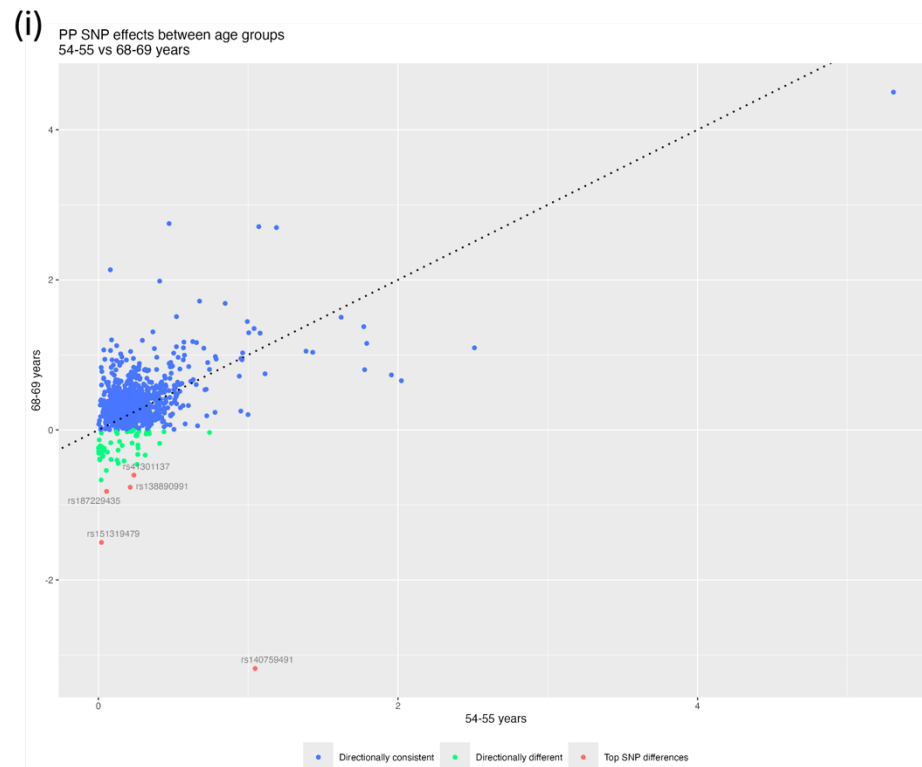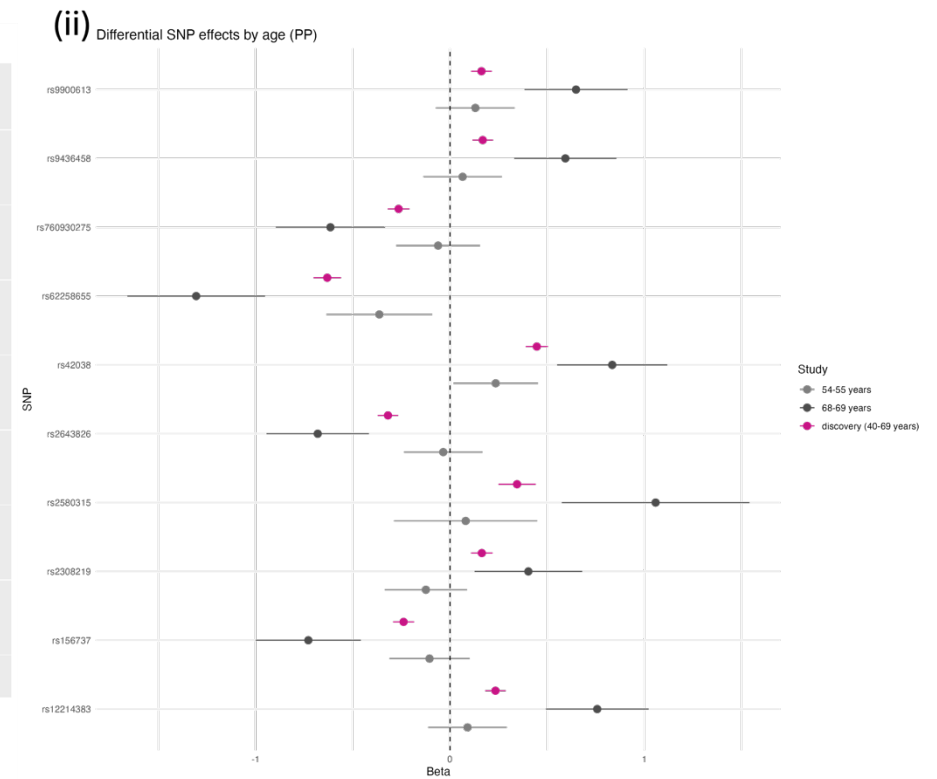

**S10 Fig Comparison of GWAS effect estimates between Stratum 8 (54-55 years) and Stratum 15 (68-69 years) for PP.**

Supplement: S10 Fig — (PDF) [file pgen.1012080.s036.pdf]

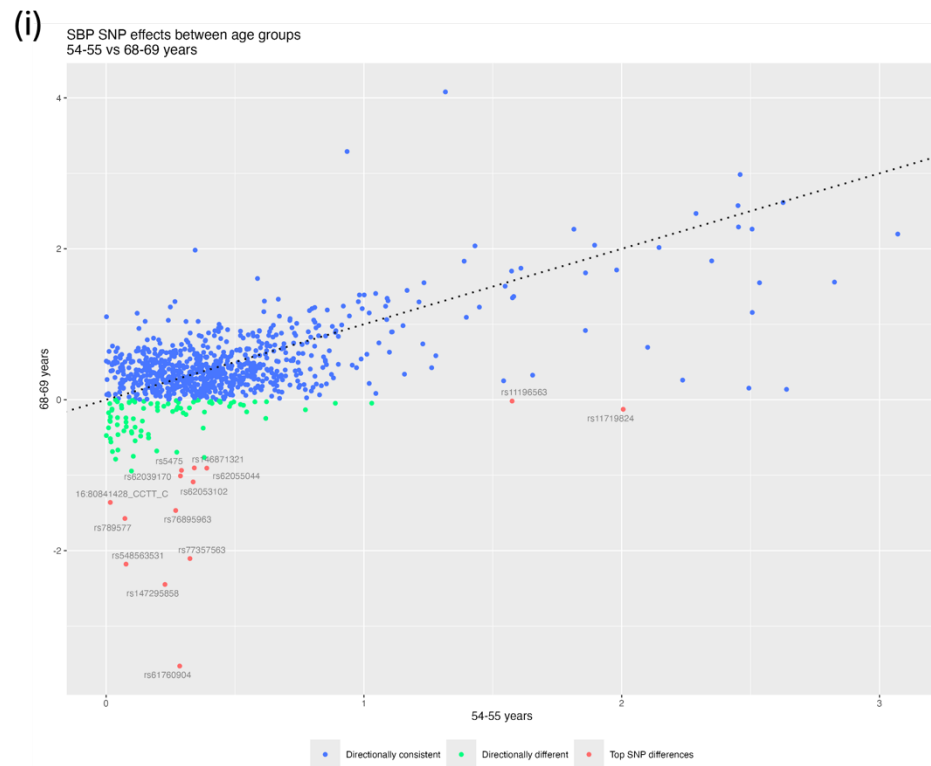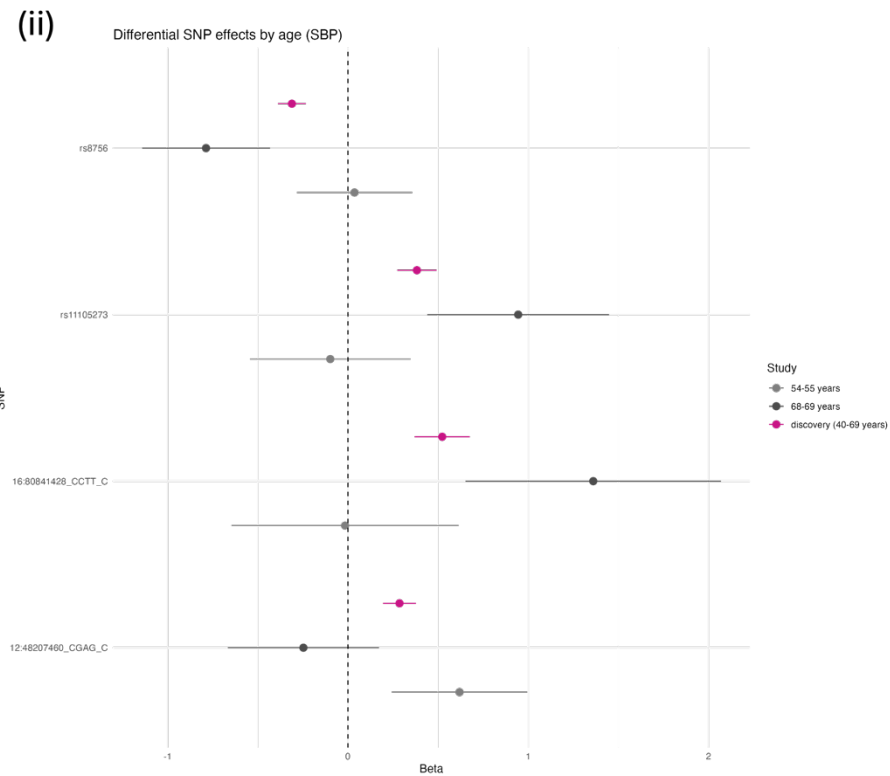

**S11 Fig Comparison of GWAS effect estimates between Stratum 8 (54-55 years) and Stratum 15 (68-69 years) for SBP.**

Supplement: S11 Fig — (PDF) [file pgen.1012080.s037.pdf]

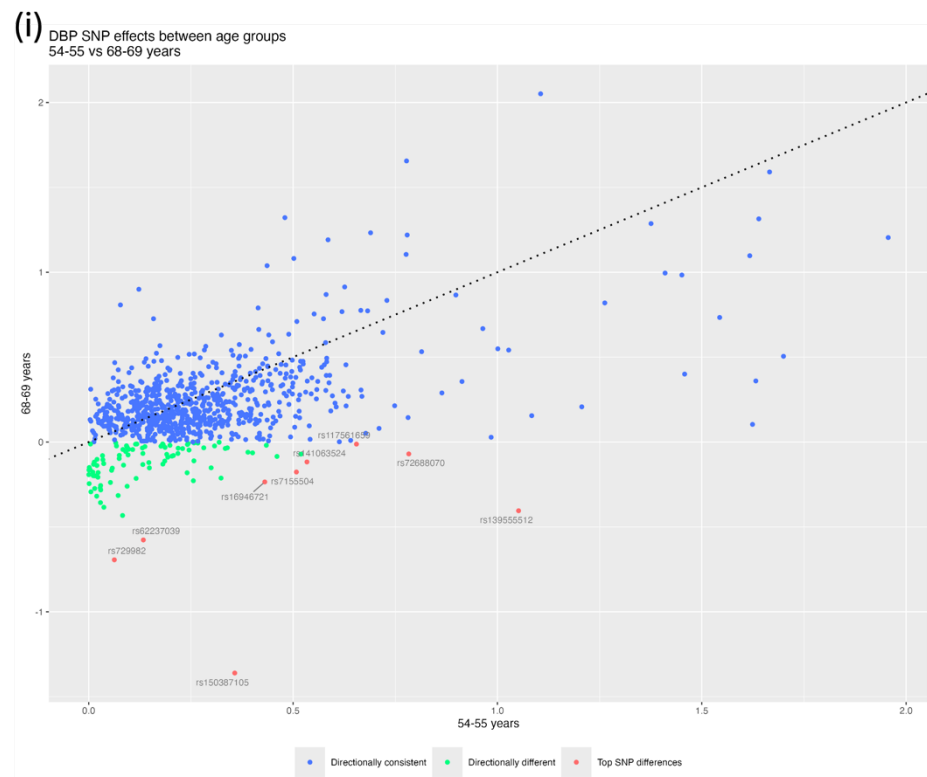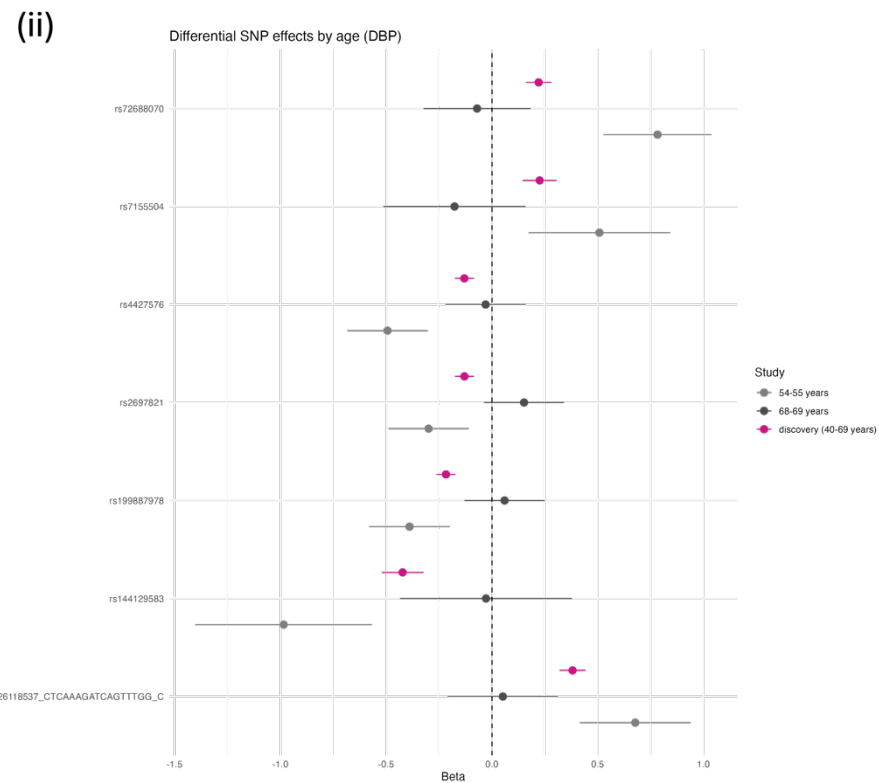

**S12 Fig Comparison of GWAS effect estimates between Stratum 8 (54-55 years) and Stratum 15 (68-69 years) for DBP.**

Supplement: S12 Fig — (PDF) [file pgen.1012080.s038.pdf]

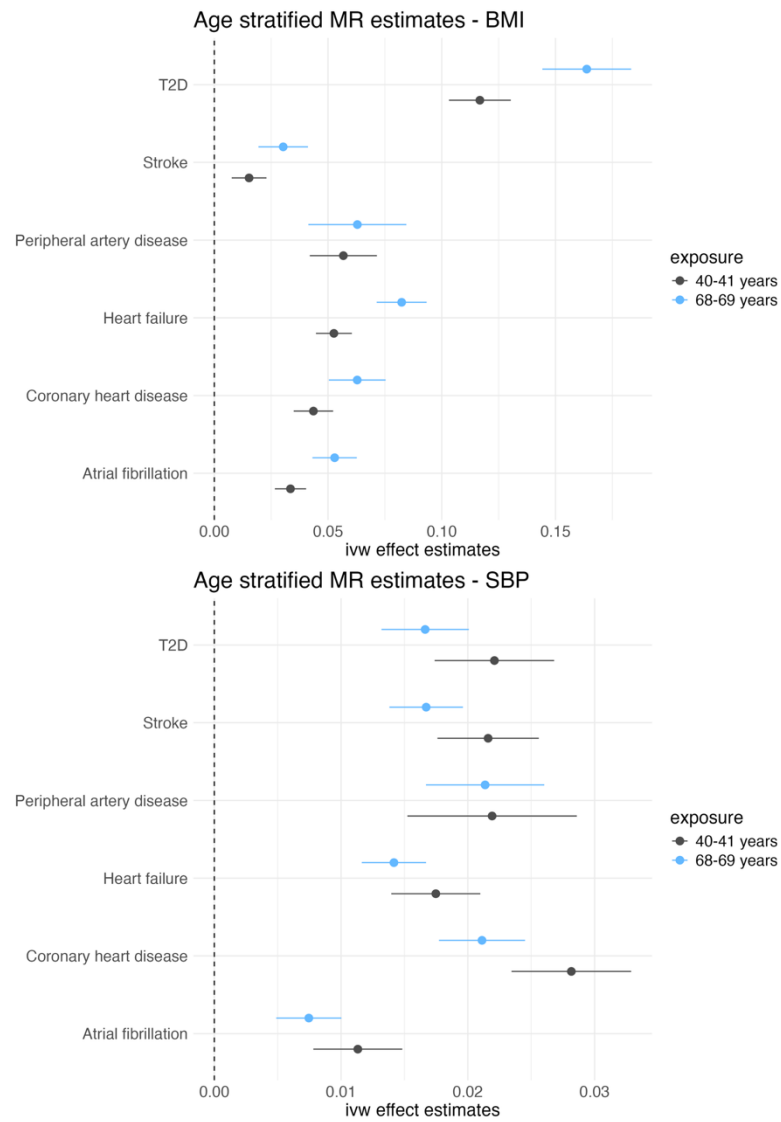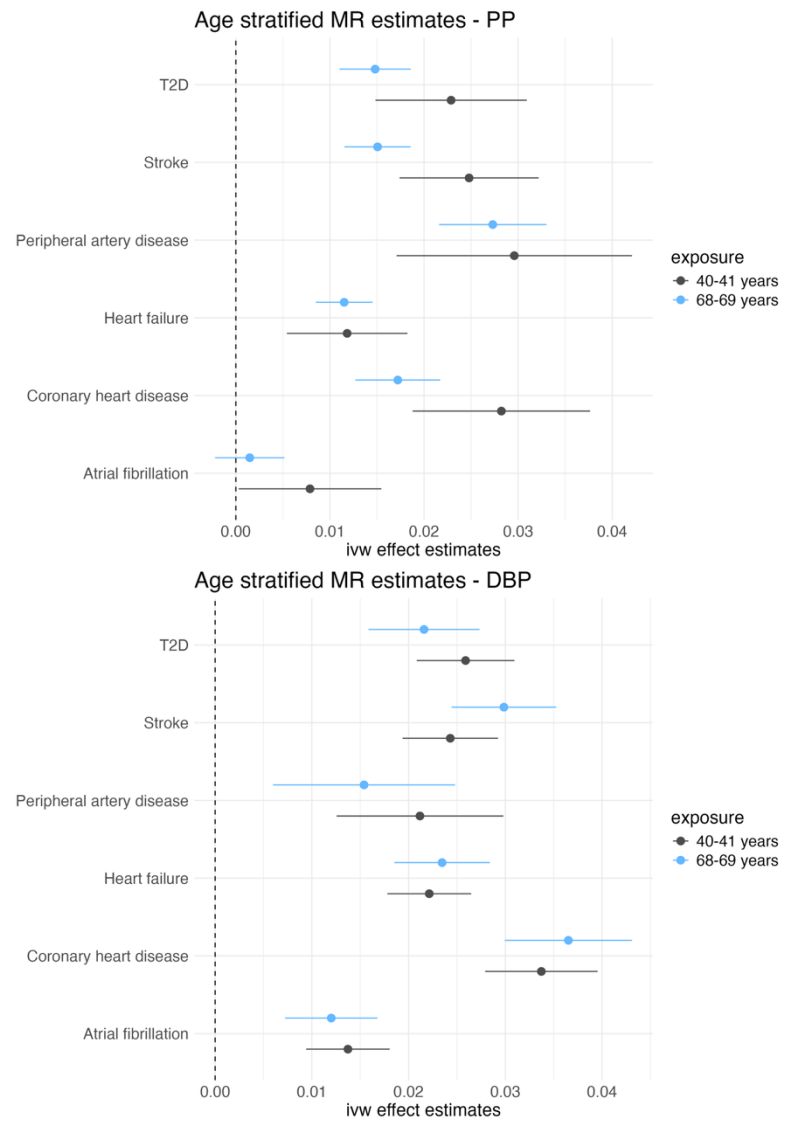

**S14 Fig Results of age-specific standard MR analyses for BMI, PP, SBP and DBP.**

Supplement: S14 Fig — (PDF) [file pgen.1012080.s042.pdf]
