# Supplementary material for: An evaluation of age-varying genetic effects underlying body-mass index and blood pressure in the UK Biobank
Source: PLoS Genet. 2026 Mar 20;22(3):e1012080. doi: 10.1371/journal.pgen.1012080 (PMC13029756; doi:10.1371/journal.pgen.1012080)
Supplement: S5 Fig — (PDF) [file pgen.1012080.s031.pdf]

Comparison 2: between youngest (age 40-41) and oldest (age 68-69) age groups.

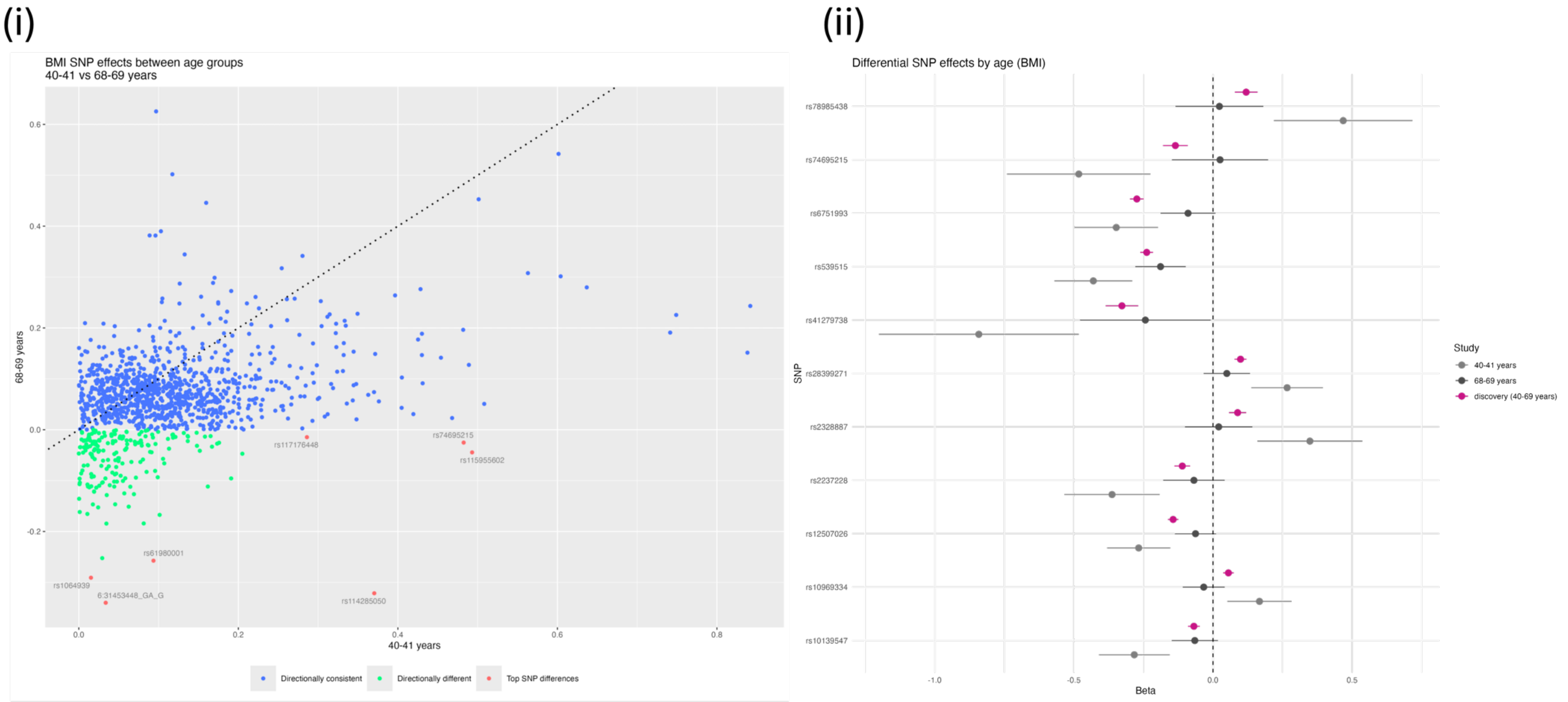

S5 Fig Comparison of GWAS effect estimates between Stratum 1 (40-41 years) and Stratum 15 (68-69 years) for BMI.
