# Supplementary material for: An evaluation of age-varying genetic effects underlying body-mass index and blood pressure in the UK Biobank
Source: PLoS Genet. 2026 Mar 20;22(3):e1012080. doi: 10.1371/journal.pgen.1012080 (PMC13029756; doi:10.1371/journal.pgen.1012080)
Supplement: S13 Fig — (PDF) [file pgen.1012080.s039.pdf]

The relationship between GWAS estimate and SNP*age interaction effects.

(i) (ii)

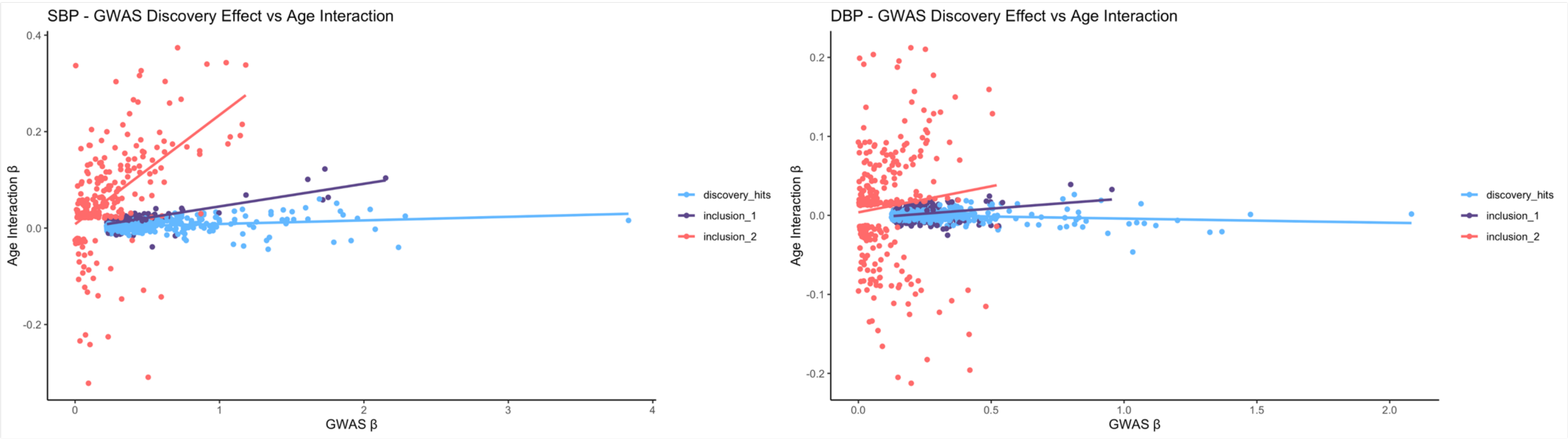

S13 Fig Scatter plot depicting the relationship between SNP-trait effect estimates derived by GWAS and SNP*age interaction effects derived by meta-regression analysis for (i) SBP and (ii) DBP. *The x-axis depicts the GWAS derived estimates, and the y-axis depicts the meta-regression derived interaction effects.*
