# Supplementary material for: An evaluation of age-varying genetic effects underlying body-mass index and blood pressure in the UK Biobank
Source: PLoS Genet. 2026 Mar 20;22(3):e1012080. doi: 10.1371/journal.pgen.1012080 (PMC13029756; doi:10.1371/journal.pgen.1012080)
Supplement: S2 Note — Table A. Summary of the number of exact SNPs identified in the sensitivity analyses using individual level data with the primary analysis. (DOCX) [file pgen.1012080.s040.docx]

## Supplementary Note 2:

## Summary of overlapping loci in sensitivity analysis

We have recorded a summary of the number of SNPs identified in our sensitivity analyses using the individual level SNP*Age model in each “inclusion group” matching the SNPs identified in our primary analysis (Table 1). The majority of variants match exactly within the discovery and inclusion 1 identified SNPs between the primary and sensitivity analysis. A proportion of the unmatched SNPs are likely to be in LD with the variants identified by using either method, due to clumping selecting a different lead variant.

**Table A. Summary of the number of exact SNPs identified in the sensitivity analyses using individual level data with the primary analysis.** Data records the number of exact SNP matches, and the percentage overlap in the sensitivity results.

| **Trait** | **Discovery** | **inclusion 1** | **inclusion 2** |
| --- | --- | --- | --- |
| BMI | 544, 57.6% | 77, 69.3% | 13, 8.38% |
| PP | 463, 63.3% | 172, 58.3% | 32, 12% |
| SBP | 490, 65.2% | 56, 50.5% | 6, 2.2% |
| DBP | 504, 67.2% | 52, 46.6% | 38, 17.4% |
