# Supplementary material for: An evaluation of age-varying genetic effects underlying body-mass index and blood pressure in the UK Biobank
Source: PLoS Genet. 2026 Mar 20;22(3):e1012080. doi: 10.1371/journal.pgen.1012080 (PMC13029756; doi:10.1371/journal.pgen.1012080)
Supplement: S3 Note — (DOCX) [file pgen.1012080.s041.docx]

## Supplementary Note 3:

## The results of age-stratified MR analyses

We conducted further *ivw* MR analyses to examine whether the exposure-outcome relationships varied depending on the age at which the exposure is measured in the study population. This was achieved by instrumenting our exposures separately using effect estimates derived within the youngest (40-41 years) and oldest (68-69 years) for our discovery SNP set. We note that the individual SNP effects derived in our age-specific GWAS are more weakly powered compared to our discovery GWAS, and results from this analysis may be considered as suggestive evidence. The results of our age specific MR analysis between individuals aged 40-41 and 68-69 produced largely consistent results across all traits. For example, the overall trend observed for BMI indicated that BMI measured in individuals aged 40-41- and 68-69-years consistently influences risk of developing each of the cardiovascular outcomes analysed except for T2D. For T2D, MR analyses conducted on the individuals in the oldest strata (68-69 years) had a larger risk estimate (OR=1.18; CI= 1.16-1.20; P= 8.99x10^-61^) compared to the risk estimate observed in the youngest strata (age 40-41 years) (OR= 1.12; CI=1.11-1.18; P= 1.42x10^-63^). Similarly, elevated PP in individuals aged 40-41 had a stronger effect on the lifetime risk of CHD (OR= 1.03; CI=1.02-1.04; P= 4.49x10^-09^) compared to individuals with higher PP aged 68-69 (OR=1.017; CI=1.013-1.022; P= 6.61x10^-14^), with a similar trend observed in SBP. Further details of these results are provided (**S15 Table and S13 Fig**).
