# Supplementary material for: An evaluation of age-varying genetic effects underlying body-mass index and blood pressure in the UK Biobank
Source: PLoS Genet. 2026 Mar 20;22(3):e1012080. doi: 10.1371/journal.pgen.1012080 (PMC13029756; doi:10.1371/journal.pgen.1012080)
Supplement: S15 Fig — (PDF) [file pgen.1012080.s043.pdf]

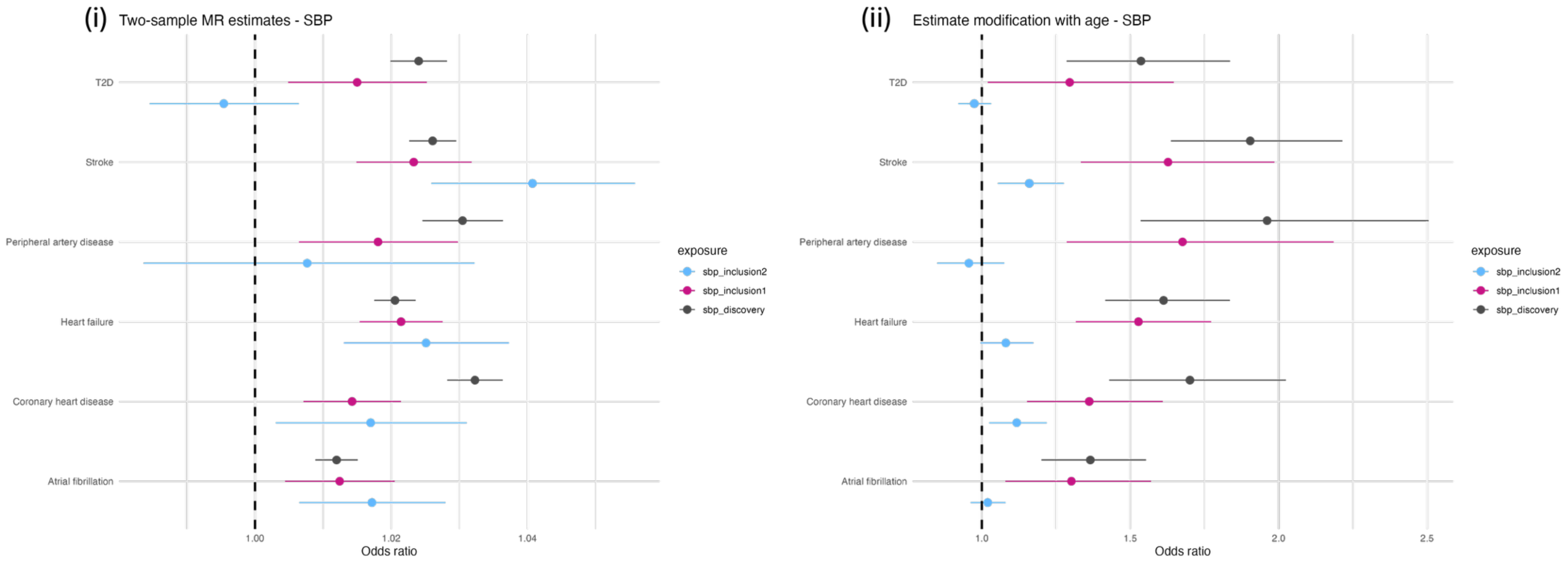

S15 Fig Standard MR estimates of (i) the effect of genetically proxied SBP measured at 40-69 years old on six cardiovascular outcomes and (ii) modified ivw estimates depicting the change in the effect of genetically proxied SBP on those outcomes per additional year of age at measurement. *Analyses were conducted separately using SNPs identified by each of our inclusion criteria (discovery, inclusion1 and inclusion2 SNPs) to instrument our exposure variables. Plots depict the log odds estimates (i) per kg m*$^{-2}$ *increase in BMI and (ii) per kg m*$^{-2}$ *per year of age. Error bars are 95% confidence intervals.*
